# U-shape association of serum albumin level and acute kidney injury risk in hospitalized patients

**DOI:** 10.1371/journal.pone.0199153

**Published:** 2018-06-21

**Authors:** Charat Thongprayoon, Wisit Cheungpasitporn, Michael A. Mao, Ankit Sakhuja, Kianoush Kashani

**Affiliations:** 1 Department of Internal Medicine, Bassett Medical Center, Cooperstown, New York, United States of America; 2 Division of Nephrology, Department of Internal Medicine, University of Mississippi Medical Center, Jackson, Mississippi, United States of America; 3 Division of Nephrology and Hypertension, Department of Medicine, Mayo Clinic, Rochester, Minnesota, United States of America; 4 Division of Pulmonary and Critical Care Medicine, Department of Medicine, Mayo Clinic, Rochester, Minnesota, United States of America; Kaohsiung Medical University Hospital, TAIWAN

## Abstract

**Background:**

While an association between hypoalbuminemia and increased risk of acute kidney injury (AKI) is well-established, the risk of AKI development and its severity among patients with elevated serum albumin is unclear. The aim of this study was to evaluate the risk of AKI in hospitalized patients stratified by various admission serum albumin levels.

**Methods:**

This single-center retrospective study was conducted at a tertiary referral hospital. All adult hospitalized patients who had admission albumin levels available between January 2009 and December 2013 were enrolled. Admission albumin was categorized based on its distribution into six groups (≤2.4, 2.5–2.9, 3.0–3.4, 3.5–3.9, 4.0–4.4, and ≥4.5 mg/dL). The primary outcome was the incidence of hospital-acquired AKI (HAKI). Logistic regression analysis was performed to obtain the odds ratio of AKI for various admission albumin strata using the albumin 3.5 to 3.9 mg/dL (lowest incidence of AKI) as the reference group.

**Results:**

Of the total 9,552 studied patients, HAKI occurred in 1,556 (16.3%) patients. The incidence of HAKI among patients with admission albumin ≤2.4, 2.5–2.9, 3.0–3.4, 3.5–3.9, 4.0–4.4, and ≥4.5 mg/dL was 18.3%, 14.3%, 15.5%, 14.2%, 16.7%, and 26.0%, respectively. After adjusting for potential confounders, admission serum albumin levels ≤2.4 and ≥4.5 mg/dL were associated with an increased risk of HAKI with odds ratios of 1.52 (95% CI 1.18–1.94) and 2.16 (95% CI 1.74–2.69), respectively. While stage 1 HAKI was significantly more frequent among patients with admission albumin ≥4.5 mg/dL (23.0% vs. 11.6%, P<0.001), incidence of stage 3 HAKI was higher in those with albumin ≤2.4 mg/dL (2.8% vs 0.3%, P<0.001).

**Conclusion:**

Admission serum albumin levels ≤2.4 and ≥4.5 mg/dL were associated with an increased risk for HAKI. Patients with admission albumin ≥4.5 mg/dL had HAKI with a lower intensity when compared with those who had admission albumin levels ≤2.4 mg/dL.

## Introduction

Acute kidney injury (AKI) is a substantial healthcare burden worldwide affecting almost 13.3 million patients per year [1, 2], associated with high morbidity and mortality, progression to chronic kidney disease (CKD), and significant healthcare costs [1–4]. AKI-related mortality has been reported to be as high as 23% or greater than 1.7 million deaths per year [1–3]. Previous studies have attempted to identify novel biomarkers of AKI, effective pharmacological interventions, and treatments to improve survival, lessen injury, or promote recovery [2, 4–10]. Most treatment strategies have been unfortunately unsuccessful [2, 7, 11]. Therefore, early identification and prevention of AKI among patients at-risk of AKI are critical.

Albumin is an important protein synthesized by the liver with multiple vital functions including osmotic pressure regulation, a carrier of poorly water-soluble molecules, antioxidant and anti-inflammatory effects [12–14]. Hypoalbuminemia is prevalent among hospitalized patients, with an incidence ranging from 16% up to 82% [12, 14–19]. Studies have shown associations of hypoalbuminemia with AKI and mortality in various clinical settings including general hospitalized patients, intensive care unit (ICU), coronary bypass surgery, emergency department, and liver transplantation [14, 15, 20–25]. Conversely, there are no studies that reported data on the incidence and effects of elevated serum albumin among patients admitted to the hospital. Thus, the objective of this study was to evaluate the risk of AKI in hospitalized patients stratified by various admission serum albumin levels.

## Materials and methods

### Study population

This is a single-center retrospective cohort study conducted at a tertiary referral hospital. All adult (≥18 years old) hospitalized patients who had admission albumin available between January 2009 and December 2013 at Mayo Clinic, Rochester, MN, USA were enrolled in this study. Exclusion criteria were patients who did not provide research authorization, those without serum albumin measurement within 24 hours of admission, individuals with end-stage renal disease (ESRD) and patients who had AKI at hospital admission. For patients with multiple admissions during this period, only the first hospitalization was analyzed. ESRD and the need for initiation of renal replacement therapy following acute kidney injury were identified based on ICD-9 (International Classification of Diseases, 9th) code assignment (S1 Table). This study was reviewed and approved by the Mayo Clinic Institutional Review Board. Informed consent was waived due to its minimal risk nature.

### Data collection

The data collection was previously described in detail in a prior study [26]. Clinical characteristics, demographic information, and laboratory data were collected using automated retrieval from the institutional electronic medical record system. The admission serum albumin level was defined as the first serum albumin level within 24 hours of hospital admission. eGFR was derived using the Chronic Kidney Disease Epidemiology Collaboration (CKD-EPI) equation [27]. CKD was defined as a calculated eGFR < 60 mL/min/1.73m^2^. The Charlson Comorbidity score [28] was computed for comorbidities at the time of admission. Principal diagnoses were grouped based on ICD-9 codes at admission (S2 Table).

### Clinical outcomes

The primary outcome was hospital-acquired AKI (HAKI), based on the KDIGO serum creatinine criterion [29]. HAKI was defined as an increase in serum creatinine of ≥ 0.3 mg/dL within 48 hours after admission date or ≥ 1.5 times baseline within 7 days after admission date. If outpatient baseline serum creatinine was not available, the Modification of Diet in Renal Disease equation [30] was used to estimate baseline serum creatinine level, assuming normal baseline GFR of 75 mL/min/1.73m^2^, in accordance with this guideline [29].

### Statistical analysis

Continuous variables are reported as mean ± SD. All categorical variables are described as counts with percentages. Baseline demographics and clinical characteristics were compared among admission serum albumin groups, using ANOVA for continuous variables and the Chi-square test for categorical variables. We categorized admission serum albumin levels, based on its distribution at 10^th^, 25^th^, 50^th^, 75^th^ and 90^th^ percentiles, into six groups (≤2.4, 2.5–2.9, 3.0–3.4, 3.5–3.9, 4.0–4.4, and ≥4.5 mg/dL). Serum albumin level 3.5 to 3.9 mg/dL, given the lowest incidence of AKI, was selected as the reference group (Table 1). We performed univariate analysis and then multivariable logistic regression analysis to assess the independent association between various admission albumin levels and HAKI. Odds ratio (OR) with 95% confidence interval (CI) are reported. OR was adjusted for a priori defined variables. The adjusting variables were age, sex, race, baseline eGFR, principal diagnosis, Charlson comorbidity score, comorbidities, medications, the need for vasopressor and mechanical ventilator at hospital admission, and alcohol use. Comorbidities were coronary artery disease (CAD), hypertension (HTN), diabetes mellitus (DM), congestive heart failure (CHF), peripheral vascular disease (PVD), stroke, and cirrhosis. Medications were angiotensin-converting-enzyme inhibitors (ACEIs), angiotensin II receptor blockers (ARBs), nonsteroidal anti-inflammatory Drugs (NSAIDs) and diuretics. A two-tailed P value of <0.05 was considered statistically significant. All analyses were performed using JMP statistical software (version 10.0, SAS Institute, Cary, NC).

**Table 1:** Baseline clinical characteristics.

Variables	All	Serum albumin level at hospital admission (mg/dl)						
		≤2.4	2.5–2.9	3.0–3.4	3.5–3.9	4.0–4.4	≥4.5	p
N	9552	672	1231	2147	2654	2086	762	
Age (year)	60±18	59±16	61±17	63±18	62±18	57±18	49±17	<0.001
Male	5028 (53)	351 (52)	652 (53)	1148 (53)	1400 (53)	1080 (52)	397 (52)	0.92
White	8613 (90)	597 (89)	1106 (90)	1943 (90)	2393 (90)	1891 (91)	683 (90)	0.77
Charlson Comorbidity Score	2.1±2.6	2.0±2.5	2.6±3.0	2.5±2.8	2.2±2.7	1.8±2.4	1.1±1.8	<0.001
GFR (ml/min/1.73m2)	87±25	93±25	90±16	86±25	85±25	86±24	92±22	<0.001
Comorbidities								
- Coronary artery disease	1576 (16)	78 (12)	192 (16)	377 (18)	519 (20)	351 (17)	59 (8)	<0.001
- Hypertension	4154 (43)	261 (39)	507 (41)	1017 (47)	1284 (48)	885 (42)	200 (26)	<0.001
- Diabetes mellitus	1752 (18)	110 (16)	253 (21)	415 (19)	552 (21)	357 (17)	65 (9)	<0.001
- Congestive heart failure	677 (7)	28 (4)	86 (7)	155 (7)	225 (8)	151 (7)	32 (4)	<0.001
- Peripheral vascular disease	238 (2)	11 (2)	46 (4)	61 (3)	65 (2)	45 (2)	10 (1)	<0.001
- Stroke	651 (7)	24 (4)	87 (7)	143 (7)	238 (9)	132 (6)	27 (4)	<0.001
- Cirrhosis	624 (7)	95 (14)	155 (13)	175 (8)	122 (5)	61 (3)	16 (2)	<0.001
Principal diagnosis								<0.001
- Cardiovascular	1579 (17)	32 (5)	133 (11)	286 (13)	560 (21)	447 (21)	121 (16)	
- Hematology/Oncology	1708 (18)	108 (16)	203 (16)	403 (19)	447 (17)	399 (19)	148 (19)	
- Infectious disease	442 (5)	85 (13)	104 (8)	124 (6)	93 (4)	28 (1)	8 (1)	
- Endocrine/Metabolic	445 (5)	28 (4)	65 (5)	105 (5)	124 (5)	102 (5)	21 (3)	
- Respiratory	561 (6)	40 (6)	92 (7)	164 (8)	154 (6)	93 (4)	18 (2)	
- Gastrointestinal/Hepatology	1699 (18)	203 (30)	313 (25)	436 (20)	436 (16)	233 (11)	78 (10)	
- Injury/Poisoning	1569 (16)	84 (13)	149 (12)	312 (15)	360 (14)	419 (20)	245 (32)	
- Other	1549 (16)	92 (14)	172 (14)	317 (15)	480 (18)	365 (17)	123 (16)_	
Urgency of admission								<0.001
- Emergent	4267 (45)	286 (43)	551 (45)	1074 (50)	1326 (50)	823 (40)	207 (27)	
- Urgent	2468 (26)	241 (36)	373 (30)	567 (26)	678 (26)	466 (22)	143 (19)	
- Elective	2817 (29)	145 (20)	307 (25)	506 (24)	650 (24)	797 (38)	412 (54)	
Surgical procedures requiring general anesthesia	3397 (36)	240 (36)	404 (33)	702 (33)	791 (30)	833 (40)	427 (56)	<0.001
Medication								
- ACEI/ARB	2878 (30)	148 (22)	305 (25)	664 (31)	896 (34)	676 (32)	189 (25)	<0.001
- Diuretics	3443 (36)	287 (43)	510 (41)	812 (38)	958 (36)	701 (34)	175 (23)	<0.001
- NSAID	1633 (17)	99 (15)	169 (14)	352 (16)	448 (17)	395 (19)	170 (22)	<0.001
Received contrast media	2970 (31)	306 (46)	490 (40)	775 (36)	821 (31)	456 (22)	122 (16)	<0.001
Received IV Fluid Bolus	1000 (10)	165 (25)	167 (14)	236 (11)	214 (8)	159 (8)	59 (8)	<0.001
Vasopressor use	459 (5)	58 (9)	72 (6)	108 (5)	97 (4)	76 (4)	49 (6)	<0.001
Mechanical ventilator	945 (10)	110 (16)	142 (12)	235 (11)	202 (8)	180 (9)	76 (10)	<0.001
Alcohol use	926 (10)	103 (15)	118 (10)	209 (10)	249 (9)	197 (9)	50 (7)	<0.001
Urine Protein (mg/dL), Median (IQR)	213 (106–463)	321 (157–668)	303 (148–617)	243 (131–509)	180-93-364)	139 (78–355)	127 (72–236)	<0.001

Continuous data are presented as mean ± SD; categorical data are presented as count (percentage)

## Results

A total of 14,075 patients with available serum albumin measurement within 24 hours were identified. Among all screened patients, 968 individuals with ESRD, 3,551 patients with AKI at admission, and 4 patients without serum creatinine measurement during hospitalization were excluded. Finally, 9,552 unique patients were enrolled in the analysis (S1 Fig).

### Baseline characteristics

The baseline characteristics of the 9,552 patients with various serum albumin levels are summarized in Table 1. The distribution of serum albumin levels was as follows: ≤2.4 mg/dL, 672 patients (7.0%); 2.5–2.9 mg/dL, 1,231 patients (12.9%); 3.0–3.4 mg/dL, 2,147 patients (22.5%); 3.5–3.9 mg/dL, 2,654 patients (27.8%); 4.0–4.4 mg/dL, 2,086 patients (21.8%); and ≥4.5 mg/dL, 762 patients (8.0%%). Of the 9,552 patients, 8,613 (90%) patients were whites and 5,028 (53%) were male. Mean (±SD) age was 60±18 years. Patient comorbidities included HTN (43%), DM (18%), CAD (16%), and CHF (7%). Prior to admission, 36% of the patients were taking diuretics, 30% were on ACEIs or ARBs, and 17% were receiving NSAIDs.

Analysis of the principle admission diagnosis showed that patients with a diagnosis of gastrointestinal tract/liver-related problems presented with low admission serum albumin levels, while patients with trauma/injury and poisoning diagnoses presented with high admission serum albumin levels (Table 1).

### Admission serum albumin levels and risk of acute kidney injury

**Fig 1:**
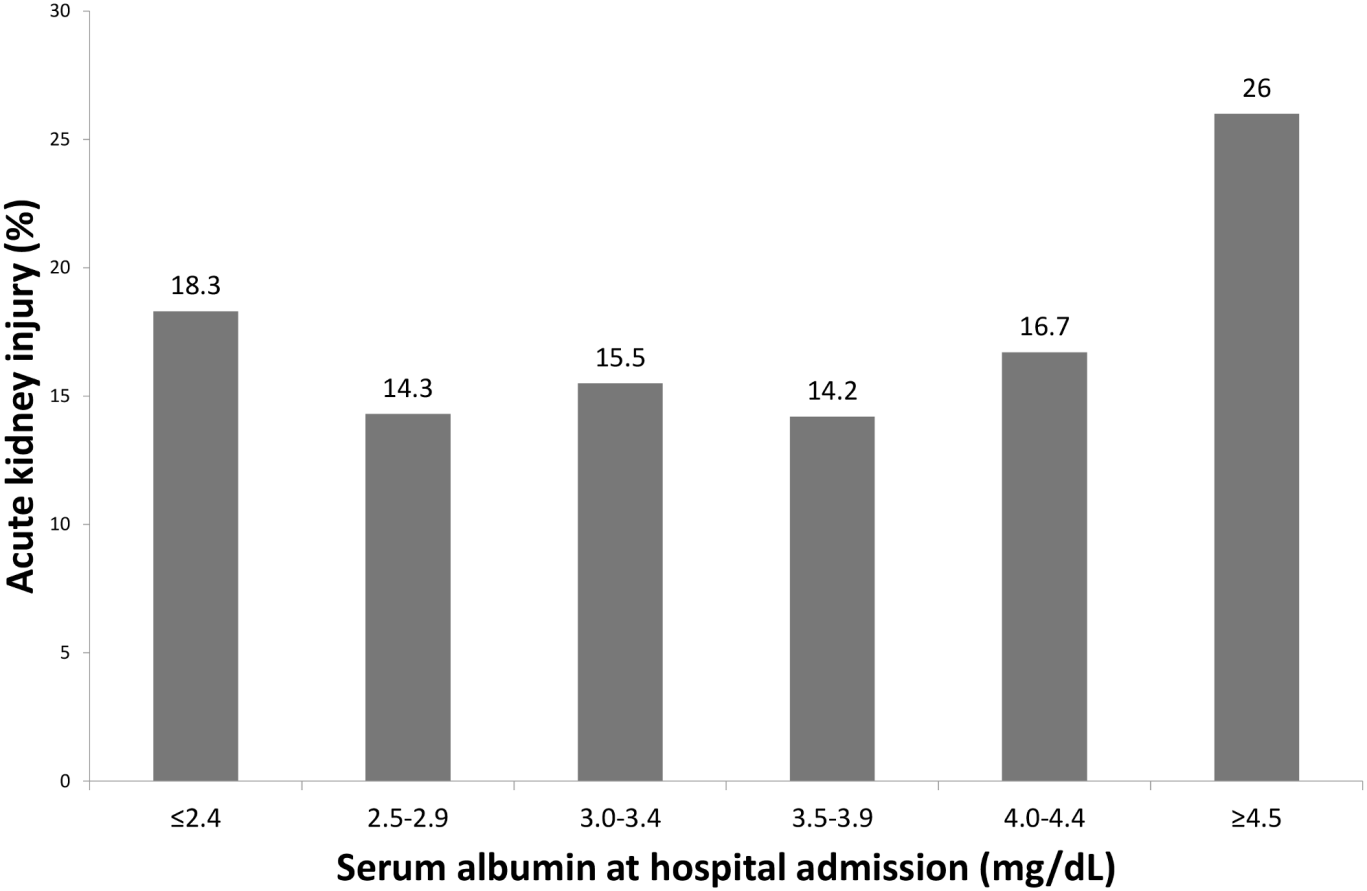
Hospital acquired acute kidney injury (HAKI) incidence with various admission serum albumin levels.

The incidence of HAKI was 18.3%, 14.3%, 15.5%, 14.2%, 16.7%, and 26.0% in patients with admission albumin ≤2.4, 2.5–2.9, 3.0–3.4, 3.5–3.9, 4.0–4.4, and ≥4.5 mg/dL, respectively (Fig 1, Table 2). Severity and HAKI staging and the incidence of renal replacement therapy initiation after HAKI among patients with various admission serum albumin levels are shown in Table 2. While the incidence of stage 1 HAKI among admission albumin ≥4.5 mg/dL group was significantly higher than the incidence among admission serum albumin ≤2.4 mg/dL group (23.0% vs. 11.6%, P<0.001), the incidence of stage 3 HAKI in admission serum albumin ≤2.4 mg/dL group was significantly higher than the incidence in admission serum albumin ≥4.5 mg/dL group (2.8% vs. 0.3%, P<0.001). This, in turn, translated to higher need for renal replacement therapy rate among patients with serum albumin ≤2.4 mg/dL when compared with patients with admission serum albumin of ≥4.5 mg/dL (3.4% vs. 0.7%, P<0.001).

**Table 2:** Hospital acquired acute kidney injury (HAKI) and mortality among patients with various admission serum albumin levels.

Outcome	All (N = 9552)	Serum albumin level at hospital admission (mg/dl)						
		≤2.4 (N = 672)	2.5–2.9 (N = 1231)	3.0–3.4 (N = 2147)	3.5–3.9 (N = 2654)	4.0–4.4 (N = 2086)	≥4.5 (N = 762)	P
HAKI	1556 (16.3)	123 (18.3)	176 (14.3)	333 (15.5)	378 (14.2)	348 (16.7)	198 (26.0)	<0.001
HAKI stage								<0.001
- Stage 1	1275 (13.3)	78 (11.6)	136 (11.0)	261 (12.2)	319 (12)	306 (14.7)	175 (23.0)	
- Stage 2	188 (2.0)	26 (3.9)	18 (1.5)	44 (2.0)	45 (1.7)	34 (1.6)	21 (2.8)	
- Stage 3	93 (1.0)	19 (2.8)	22 (1.8)	28 (1.3)	14 (0.5)	8 (0.4)	2 (0.3)	
Need for renal replacement therapy	119 (1.3)	23 (3.4)	24 (2.0)	27 (1.3)	30 (1.1)	10 (0.5)	5 (0.7)	<0.001
In-hospital mortality	247 (2.6)	52 (7.7)	43 (3.5)	66 (3.1)	63 (2.4)	17 (0.8)	6 (0.8)	<0.001

Among 9,552 patients, 247 (2.6%) died in the hospital. The lowest in-hospital mortality (0.8%) was observed in patients with admission serum albumin of ≥4.0 mg/dL (Table 2). The in-hospital mortality progressively increased with lower levels of admission serum albumin. The highest crude in-hospital mortality was observed in patients with admission serum albumin ≤2.4 mg/dL.

To assess whether admission serum albumin levels were independently associated with HAKI development, logistic regression models were built, using 3.5–3.9 mg/dL (lowest incidence of HAKI) as a reference range. In unadjusted analysis, an admission albumin ≤2.4 mg/dL,4.0–4.4 mg/dL, and ≥4.5 mg/dL were associated with an increased risk of HAKI with odds ratios of 1.35 (95% CI 1.07–1.68), 1.21 (95%CI 1.03–1.41), and 2.11 (95%CI 1.74–2.57), respectively. When adjusted for potential confounders, admission albumin ≤2.4 mg/dL and ≥4.5 mg/dL were associated with an increased risk of HAKI with odds ratios of 1.52 (95% CI 1.18–1.94) and 2.16 (95%CI 1.74–2.69), respectively (Table 3). Admission serum albumin levels between 2.5 and 4.4 mg/dL were not predictive for the development of HAKI during hospitalization.

**Table 3:** Odds ratios for the association between admission serum albumin levels and hospital acquired acute kidney injury (HAKI) occurrence.

Serum albumin level at hospital admission (mg/dl)	Univariate analysis		Multivariate analysis	
	OR (95% CI)	p	Adjusted OR (95% CI)	p
≤2.4	1.35 (1.07–1.68)	0.01	1.52 (1.18–1.94)	0.001
2.5–2.9	1.004 (0.83–1.22)	0.96	1.05 (0.85–1.29)	0.65
3.0–3.4	1.11 (0.94–1.30)	0.21	1.10 (0.93–1.31)	0.27
3.5–3.9	1 (ref)		1 (ref)	
4.0–4.4	1.21 (1.03–1.41)	0.02	1.18 (0.99–1.39)	0.06
≥4.5	2.11 (1.74–2.57)	<0.001	2.16 (1.74–2.69)	<0.001

Adjusted for age, sex, race, Charlson Comorbidity score, baseline GFR, history of coronary artery disease, hypertension, diabetes mellitus, congestive heart failure, peripheral vascular disease, stroke, cirrhosis, principal diagnosis, use of ACEI/ARB, NSAID, diuretics, the need for vasopressor and mechanical ventilator at hospital admission, alcohol use

### Subgroup analysis based on cirrhosis and chronic kidney disease

Of the 9,552 patients, 624 patients had cirrhosis. After adjusting for potential confounders, admission serum albumin ≤2.4 mg/dL were associated with an increased risk of HAKI in patients with cirrhosis with adjusted OR of 2.60 (95% CI 1.32–5.21) (Table 4). In analyses of 8,928 non-cirrhotic patients, admission serum albumin ≤2.4 and ≥ 4.5 mg/dL were associated with an increased risk of HAKI with adjusted odds ratios of 1.34 (95% CI 1.01–1.76) and 2.19 (95%CI 1.75–2.74), respectively (Table 4).

**Table 4:** Odds ratios for the association between admission serum albumin levels and hospital acquired acute kidney injury (HAKI) occurrence in subgroups of patients with and without cirrhosis.

Serum albumin level at hospital admission (mg/dl)	Univariate analysis		Multivariate analysis	
	OR (95% CI)	p	Adjusted OR (95% CI)	p
Cirrhosis (n = 624)				
≤2.4	1.87 (1.04–3.37)	0.03	2.60 (1.32–5.21)	0.006
2.5–2.9	0.83 (0.47–1.46)	0.52	1.07 (0.56–2.02)	0.84
3.0–3.4	1.16 (0.69–1.98)	0.58	1.07 (0.59–1.97)	0.82
3.5–3.9	1 (ref)		1 (ref)	
4.0–4.4	0.60 (0.26–1.29)	0.20	0.79 (0.32–1.87)	0.60
≥4.5	0.44 (0.07–1.69)	0.25	0.53 (0.07–2.32)	0.42
Without cirrhosis (n = 8928)				
≤2.4	1.11 (0.86–1.43)	0.41	1.34 (1.01–1.76)	0.04
2.5–2.9	0.96 (0.78–1.18)	0.72	1.05 (0.83–1.31)	0.68
3.0–3.4	1.06 (0.90–1.26)	0.50	1.09 (0.91–1.31)	0.34
3.5–3.9	1 (ref)		1 (ref)	
4.0–4.4	1.26 (1.07–1.48)	0.006	1.19 (0.99–1.41)	0.06
≥4.5	2.24 (1.83–2.73)	<0.001	2.19 (1.75–2.74)	<0.001

Adjusted for age, sex, race, Charlson Comorbidity score, baseline GFR, history of coronary artery disease, hypertension, diabetes mellitus, congestive heart failure, peripheral vascular disease, stroke, principal diagnosis, use of ACEI/ARB, NSAID, diuretics, the need for vasopressor and mechanical ventilator at hospital admission, alcohol use

Within the main cohort, 959 patients had chronic kidney disease. In this subgroup, there was no significant association between admission serum albumin and HAKI (Table 5).

**Table 5:** Odds ratios for the association between admission serum albumin levels and hospital acquired acute kidney injury (HAKI) occurrence in subgroups of patients with chronic kidney disease (n = 959).

Serum albumin level at hospital admission (mg/dl)	Univariate analysis		Multivariate analysis	
	OR (95% CI)	p	Adjusted OR (95% CI)	p
≤2.4	2.06 (0.97–4.23)	0.06	2.17 (0.93–4.88)	0.07
2.5–2.9	1.00 (0.57–1.71)	0.99	1.08 (0.59–1.93)	0.79
3.0–3.4	1.23 (0.84–1.81)	0.29	1.21 (0.80–1.83)	0.37
3.5–3.9	1 (ref)		1 (ref)	
4.0–4.4	1.06 (0.70–1.59)	0.78	1.12 (0.72–1.74)	0.61
≥4.5	0.77 (0.32–1.66)	0.52	0.84 (0.33–1.92)	0.69

Adjusted for age, sex, race, Charlson Comorbidity score, baseline GFR, history of coronary artery disease, hypertension, diabetes mellitus, congestive heart failure, peripheral vascular disease, stroke, cirrhosis, principal diagnosis, use of ACEI/ARB, NSAID, diuretics, the need for vasopressor and mechanical ventilator at hospital admission, alcohol use

## Discussion

In this large retrospective cohort study, we demonstrated that admission serum albumin levels ≤2.4 and ≥4.5 mg/dL were associated with an increased risk for HAKI. Admission serum albumin levels ≥4.5 mg/dL were associated with the highest risk (2.16-fold) of HAKI. However, compared with serum albumin levels ≤2.4 mg/dL, the severity of HAKI among patients with serum albumin ≥4.5 mg/dL was significantly lower. Among patients with cirrhosis, only admission serum albumin level ≤2.4 mg/dL was associated with risk of HAKI (2.60-fold).

Several factors can influence hypoalbuminemia including inflammation and/or infections, malnutrition and/or protein-losing disorders, oxidative stress, cancer cachexia, and liver dysfunction [31–37]. Thus, hypoalbuminemia may indicate the severity of an underlying disease and/or a marker of malnutrition [13, 38]. In addition, studies have also consistently demonstrated that hypoalbuminemia is independently associated with increased risks of AKI development and mortality in critically ill, among various surgical and other settings [14, 15, 20–25]. In addition to the albumin role in maintenance of plasma volume by preserving colloid osmotic pressure, it also provides many physiological effects, including coupling and carrying various endogenous and exogenous toxic substances, scavenging free radicals, maintaining capillary membrane permeability, providing a physiological reservoir of nitric oxide, imparting an anti-inflammatory effect, and finally inhibition of apoptosis [15, 38, 39]. Several studies have suggested that serum albumin can preserve the kidneys from toxic agents and maintain optimal oncotic pressure and kidney perfusion [40–45]. In multivariate analysis adjusted for potential confounders, we confirmed an association between admission hypoalbuminemia and an increased risk of AKI among general hospitalized patients.

Our study is the first to demonstrate an association between elevated admission serum albumin ≥4.5 mg/dL and an increased risk of HAKI. Elevated serum albumin levels have been described in patients with dehydration and high protein diet consumption [46–48]. Although high-protein diets can increase albumin synthesis [49], albumin only increases by small increments, and high serum albumin levels ≥4.5 mg/dL are mostly caused by volume depletion [46–48]. Thus, intravascular volume depletion likely explains the association between elevated admission serum albumin and an increased risk of HAKI in our study. This likely explains why the HAKI severity in the setting of elevated serum albumin ≥4.5 mg/dL is lower, compared with HAKI occurring in patients with serum albumin levels ≤2.4 mg/dL who may have more severe underlying illness. The findings from our study demonstrated that hospital mortality did not reflect the U-shaped association as seen with AKI incidence. On the contrary, death was lowest for those with serum albumin levels ≥4 mg/dL. Additionally, these findings support our assumption that increased rates of lower stages of AKI among patients with serum albumin levels ≥4.5 mg/dL are mostly due to volume depletion and of minor clinical relevance.

Although there is potential evidence that use of intravenous albumin may reduce the risk of AKI in particular patient population, such as off-pump coronary artery bypass surgery (CABG) patients [50], it is unclear if normalization of admission serum albumin can reduce the risk of in-hospital AKI among general hospitalized patients. In a meta-analysis of 17 randomized controlled trials assessing the effect of albumin as a resuscitation fluid for patients with sepsis [51], the use of albumin-containing solutions was associated with lower mortality compared with other fluid resuscitation regimens. Lee et al. [50] recently conducted a trial assessing effect of exogenous albumin on the incidence of postoperative AKI in patients undergoing off-pump CABG with a preoperative albumin level <4.0 g/dL and demonstrated that 20% exogenous albumin administration immediately before the operation increased urine output during surgery and decreased the risk of postoperative AKI [50]. A recent retrospective cohort study by Yu et al. suggested that replacement of albumin after the development of AKI may also promote renal recovery [14]. In a meta-analysis of eleven randomized clinical trials evaluating the effect of hyperoncotic colloids on AKI in a wide variety of clinical scenarios including ascites, surgery, sepsis and spontaneous bacterial peritonitis, Wiedermann et al. demonstrated that administration of hyperoncotic albumin solutions could decrease the odds of AKI by 76% [52]. Thus, future prospective trials assessing the potentially beneficial effect of intravenous albumin administration on the risk of AKI among general hospitalized patients are warranted.

There are some limitations that bear mentioning. First, this is a single-center, historical cohort study. The patient population in our cohort is comparatively homogeneous consisting of predominantly whites. Future studies with a more diversified population and more comprehensive clinical information are needed to better assess the effects of admission serum albumin levels on HAKI. Second, the number of patients with the diagnosis of cirrhosis was small (N = 624). The failure to observe any association between elevated admission serum albumin levels ≥4.5 mg/dL and HAKI in patients with cirrhosis could be due to small sample size (Type II error). Lastly, based on the retrospective nature of this design a causal relationship could not be inferred.

In summary, this study demonstrates that admission serum albumin levels ≤2.4 and ≥4.5 mg/dL are associated with an increased risk for in-hospital AKI. However, AKI in patients with admission serum albumin ≥4.5 mg/dL is less severe and has a lower association with higher mortality rates, compared to those with serum albumin levels ≤2.4 mg/dL.

## Supporting information

S1 Table ICD-9 for end-stage renal disease. (DOCX)

S2 Table ICD-9 for principal diagnosis. (DOCX)

S1 Fig Study flow. (DOCX)

S1 Dataset Dataset. (JMP)

## References

[1] Mehta RL, Cerda J, Burdmann EA, Tonelli M, Garcia-Garcia G, Jha V, et al International Society of Nephrology’s 0by25 initiative for acute kidney injury (zero preventable deaths by 2025): a human rights case for nephrology. Lancet. 2015;385(9987):2616–43. doi: 10.1016/S0140-6736(15)60126-X . 25777661 10.1016/S0140-6736(15)60126-X

[2] Zuk A, Bonventre JV. Acute Kidney Injury. Annual review of medicine. 2016;67:293–307. Epub 2016/01/16. doi: 10.1146/annurev-med-050214-013407 . 26768243 10.1146/annurev-med-050214-013407 PMC4845743

[3] Thongprayoon C, Cheungpasitporn W, Akhoundi A, Ahmed AH, Kashani KB. Actual versus ideal body weight for acute kidney injury diagnosis and classification in critically Ill patients. BMC Nephrol. 2014;15(1):176 Epub 2014/11/16. doi: 10.1186/1471-2369-15-176 . 25398596 10.1186/1471-2369-15-176 PMC4236495

[4] Kashani K, Cheungpasitporn W, Ronco C. Biomarkers of acute kidney injury: the pathway from discovery to clinical adoption. Clinical chemistry and laboratory medicine. 2017;55(8):1074–89. Epub 2017/01/12. doi: 10.1515/cclm-2016-0973 . 28076311 10.1515/cclm-2016-0973

[5] Cheng X, Tong J, Hu Q, Chen S, Yin Y, Liu Z. Meta-analysis of the effects of preoperative renin-angiotensin system inhibitor therapy on major adverse cardiac events in patients undergoing cardiac surgery. Eur J Cardiothorac Surg. 2014 Epub 2014/10/11. doi: 10.1093/ejcts/ezu330 . 25301954 10.1093/ejcts/ezu330

[6] Yacoub R, Patel N, Lohr JW, Rajagopalan S, Nader N, Arora P. Acute kidney injury and death associated with renin angiotensin system blockade in cardiothoracic surgery: a meta-analysis of observational studies. Am J Kidney Dis. 2013;62(6):1077–86. Epub 2013/06/25. doi: 10.1053/j.ajkd.2013.04.018 . 23791246 10.1053/j.ajkd.2013.04.018

[7] Garg AX, Kurz A, Sessler DI, Cuerden M, Robinson A, Mrkobrada M, et al Perioperative Aspirin and Clonidine and Risk of Acute Kidney Injury: A Randomized Clinical Trial. JAMA. 2014 Epub 2014/11/16. doi: 10.1001/jama.2014.15284 . 25399007 10.1001/jama.2014.15284

[8] Parikh CR, Moledina DG, Coca SG, Thiessen-Philbrook HR, Garg AX. Application of new acute kidney injury biomarkers in human randomized controlled trials. Kidney international. 2016;89(6):1372–9. Epub 2016/05/12. doi: 10.1016/j.kint.2016.02.027 . 27165835 10.1016/j.kint.2016.02.027 PMC4869991

[9] Kashani K, Cheungpasitporn W, Ronco C. Biomarkers of acute kidney injury: the pathway from discovery to clinical adoption. Clin Chem Lab Med. 2017 doi: 10.1515/cclm-2016-0973 . 28076311 10.1515/cclm-2016-0973

[10] Thongprayoon C, Cheungpasitporn W, Gillaspie EA, Greason KL, Kashani KB. The risk of acute kidney injury following transapical versus transfemoral transcatheter aortic valve replacement: a systematic review and meta-analysis. Clin Kidney J. 2016;9(4):560–6. doi: 10.1093/ckj/sfw055 . 27478597 10.1093/ckj/sfw055 PMC4957730

[11] Wilson FP, Shashaty M, Testani J, Aqeel I, Borovskiy Y, Ellenberg SS, et al Automated, electronic alerts for acute kidney injury: a single-blind, parallel-group, randomised controlled trial. Lancet. 2015;385(9981):1966–74. Epub 2015/03/03. doi: 10.1016/S0140-6736(15)60266-5 . 25726515 10.1016/S0140-6736(15)60266-5 PMC4475457

[12] Namendys-Silva SA, Gonzalez-Herrera MO, Texcocano-Becerra J, Herrera-Gomez A. Hypoalbuminemia in critically ill patients with cancer: incidence and mortality. The American journal of hospice & palliative care. 2011;28(4):253–7. Epub 2010/11/09. doi: 10.1177/1049909110384841 . 21057142 10.1177/1049909110384841

[13] Vincent JL. Relevance of albumin in modern critical care medicine. Best practice & research Clinical anaesthesiology. 2009;23(2):183–91. Epub 2009/08/06. . 19653438 10.1016/j.bpa.2008.11.004

[14] Yu MY, Lee SW, Baek SH, Na KY, Chae DW, Chin HJ, et al Hypoalbuminemia at admission predicts the development of acute kidney injury in hospitalized patients: A retrospective cohort study. PloS one. 2017;12(7):e0180750 Epub 2017/07/21. doi: 10.1371/journal.pone.0180750 . 28723973 10.1371/journal.pone.0180750 PMC5516984

[15] Wiedermann CJ, Wiedermann W, Joannidis M. Hypoalbuminemia and acute kidney injury: a meta-analysis of observational clinical studies. Intensive Care Med. 2010;36(10):1657–65. doi: 10.1007/s00134-010-1928-z . 20517593 10.1007/s00134-010-1928-z PMC7728653

[16] Sung J, Bochicchio GV, Joshi M, Bochicchio K, Costas A, Tracy K, et al Admission serum albumin is predicitve of outcome in critically ill trauma patients. The American surgeon. 2004;70(12):1099–102. Epub 2005/01/25. . 15663053

[17] Fleck A, Raines G, Hawker F, Trotter J, Wallace PI, Ledingham IM, et al Increased vascular permeability: a major cause of hypoalbuminaemia in disease and injury. Lancet. 1985;1(8432):781–4. Epub 1985/04/06. . 2858667 10.1016/s0140-6736(85)91447-3

[18] Blunt MC, Nicholson JP, Park GR. Serum albumin and colloid osmotic pressure in survivors and nonsurvivors of prolonged critical illness. Anaesthesia. 1998;53(8):755–61. Epub 1998/11/03. . 9797519 10.1046/j.1365-2044.1998.00488.x

[19] Reinhardt GF, Myscofski JW, Wilkens DB, Dobrin PB, Mangan JE Jr., Stannard RT. Incidence and mortality of hypoalbuminemic patients in hospitalized veterans. JPEN Journal of parenteral and enteral nutrition. 1980;4(4):357–9. Epub 1980/07/01. doi: 10.1177/014860718000400404 . 6774116 10.1177/014860718000400404

[20] Sang BH, Bang JY, Song JG, Hwang GS. Hypoalbuminemia Within Two Postoperative Days Is an Independent Risk Factor for Acute Kidney Injury Following Living Donor Liver Transplantation: A Propensity Score Analysis of 998 Consecutive Patients. Critical care medicine. 2015;43(12):2552–61. Epub 2015/08/27. doi: 10.1097/CCM.0000000000001279 . 26308436 10.1097/CCM.0000000000001279

[21] Liu M, Chan CP, Yan BP, Zhang Q, Lam YY, Li RJ, et al Albumin levels predict survival in patients with heart failure and preserved ejection fraction. European journal of heart failure. 2012;14(1):39–44. Epub 2011/12/14. doi: 10.1093/eurjhf/hfr154 . 22158777 10.1093/eurjhf/hfr154

[22] Lee EH, Baek SH, Chin JH, Choi DK, Son HJ, Kim WJ, et al Preoperative hypoalbuminemia is a major risk factor for acute kidney injury following off-pump coronary artery bypass surgery. Intensive Care Med. 2012;38(9):1478–86. Epub 2012/05/24. doi: 10.1007/s00134-012-2599-8 . 22618092 10.1007/s00134-012-2599-8

[23] Asher V, Lee J, Bali A. Preoperative serum albumin is an independent prognostic predictor of survival in ovarian cancer. Medical oncology (Northwood, London, England). 2012;29(3):2005–9. Epub 2011/07/08. doi: 10.1007/s12032-011-0019-5 . 21735143 10.1007/s12032-011-0019-5

[24] Lyons O, Whelan B, Bennett K, O’Riordan D, Silke B. Serum albumin as an outcome predictor in hospital emergency medical admissions. European journal of internal medicine. 2010;21(1):17–20. Epub 2010/02/04. doi: 10.1016/j.ejim.2009.10.010 . 20122607 10.1016/j.ejim.2009.10.010

[25] Jellinge ME, Henriksen DP, Hallas P, Brabrand M. Hypoalbuminemia is a strong predictor of 30-day all-cause mortality in acutely admitted medical patients: a prospective, observational, cohort study. PloS one. 2014;9(8):e105983 Epub 2014/08/26. doi: 10.1371/journal.pone.0105983 . 25148079 10.1371/journal.pone.0105983 PMC4141840

[26] Thongprayoon C, Cheungpasitporn W, Mao MA, Sakhuja A, Erickson SB. Admission hyperphosphatemia increases the risk of acute kidney injury in hospitalized patients. J Nephrol. 2018;31(2):241–7. Epub 2017/10/05. doi: 10.1007/s40620-017-0442-6 . 28975589 10.1007/s40620-017-0442-6

[27] Levey AS, Stevens LA, Schmid CH, Zhang YL, Castro AF 3rd, Feldman HI, et al A new equation to estimate glomerular filtration rate. Ann Intern Med. 2009;150(9):604–12. . 19414839 10.7326/0003-4819-150-9-200905050-00006 PMC2763564

[28] Charlson M, Szatrowski TP, Peterson J, Gold J. Validation of a combined comorbidity index. J Clin Epidemiol. 1994;47(11):1245–51. Epub 1994/11/01. . 7722560 10.1016/0895-4356(94)90129-5

[29] Group. KDIGOKAKIW. KDIGO Clinical Practice Guidelines for Acute Kidney Injury. Kidney international. 2012;suppl 2(1):1–138.

[30] Zavada J, Hoste E, Cartin-Ceba R, Calzavacca P, Gajic O, Clermont G, et al A comparison of three methods to estimate baseline creatinine for RIFLE classification. Nephrol Dial Transplant. 2010;25(12):3911–8. Epub 2010/01/27. doi: 10.1093/ndt/gfp766 . 20100732 10.1093/ndt/gfp766

[31] Doweiko JP, Nompleggi DJ. Role of albumin in human physiology and pathophysiology. JPEN Journal of parenteral and enteral nutrition. 1991;15(2):207–11. Epub 1991/03/01. doi: 10.1177/0148607191015002207 . 2051560 10.1177/0148607191015002207

[32] Don BR, Kaysen G. Serum albumin: relationship to inflammation and nutrition. Seminars in dialysis. 2004;17(6):432–7. Epub 2005/01/22. doi: 10.1111/j.0894-0959.2004.17603.x . 15660573 10.1111/j.0894-0959.2004.17603.x

[33] Marrero JA, Kudo M, Bronowicki JP. The challenge of prognosis and staging for hepatocellular carcinoma. The oncologist. 2010;15 Suppl 4:23–33. Epub 2010/12/09. doi: 10.1634/theoncologist.2010-S4-23 . 21115578 10.1634/theoncologist.2010-S4-23

[34] Gupta D, Lis CG. Pretreatment serum albumin as a predictor of cancer survival: a systematic review of the epidemiological literature. Nutrition journal. 2010;9:69 Epub 2010/12/24. doi: 10.1186/1475-2891-9-69 . 21176210 10.1186/1475-2891-9-69 PMC3019132

[35] Berbel MN, Pinto MP, Ponce D, Balbi AL. Nutritional aspects in acute kidney injury. Revista da Associacao Medica Brasileira (1992). 2011;57(5):600–6. Epub 2011/10/21. . 22012298 10.1590/s0104-42302011000500022

[36] Pulimood TB, Park GR. Debate: Albumin administration should be avoided in the critically ill. Critical care (London, England). 2000;4(3):151–5. Epub 2001/02/24. doi: 10.1186/cc688 . 11211856 10.1186/cc688 PMC137253

[37] Wilkes MM, Navickis RJ. Patient survival after human albumin administration. A meta-analysis of randomized, controlled trials. Ann Intern Med. 2001;135(3):149–64. Epub 2001/08/07. . 11487482 10.7326/0003-4819-135-3-200108070-00007

[38] Nie S, Tang L, Zhang W, Feng Z, Chen X. Are There Modifiable Risk Factors to Improve AKI? BioMed research international. 2017;2017:5605634 Epub 2017/07/27. doi: 10.1155/2017/5605634 . 28744467 10.1155/2017/5605634 PMC5514336

[39] Margarson MP, Soni N. Serum albumin: touchstone or totem? Anaesthesia. 1998;53(8):789–803. Epub 1998/11/03. . 9797524 10.1046/j.1365-2044.1998.00438.x

[40] Contreras AM, Ramirez M, Cueva L, Alvarez S, de Loza R, Gamba G. Low serum albumin and the increased risk of amikacin nephrotoxicity. Revista de investigacion clinica; organo del Hospital de Enfermedades de la Nutricion. 1994;46(1):37–43. Epub 1994/01/01. . 8079062

[41] Pockaj BA, Yang JC, Lotze MT, Lange JR, Spencer WF, Steinberg SM, et al A prospective randomized trial evaluating colloid versus crystalloid resuscitation in the treatment of the vascular leak syndrome associated with interleukin-2 therapy. Journal of immunotherapy with emphasis on tumor immunology: official journal of the Society for Biological Therapy. 1994;15(1):22–8. Epub 1994/01/01. . 8110727 10.1097/00002371-199401000-00003

[42] Iglesias J, Abernethy VE, Wang Z, Lieberthal W, Koh JS, Levine JS. Albumin is a major serum survival factor for renal tubular cells and macrophages through scavenging of ROS. The American journal of physiology. 1999;277(5 Pt 2):F711–22. Epub 1999/11/24. . 10564234 10.1152/ajprenal.1999.277.5.F711

[43] Burleson RL, Jones DB, Yenikomshian AM, Cornwall C, DeVoe C, DeRito J. Clinical renal preservation by cryoperfusion with an albumin perfusate: renal perfusion with albumin. Archives of surgery (Chicago, Ill: 1960). 1978;113(6):688–92. Epub 1978/06/01. . 350192 10.1001/archsurg.1978.01370180030003

[44] Gerkens JF. Reproducible vasodilatation by platelet-activating factor in blood- and Krebs-perfused rat kidneys is albumin-dependent. European journal of pharmacology. 1990;177(3):119–26. Epub 1990/02/27. . 2311673 10.1016/0014-2999(90)90261-4

[45] Zamlauski-Tucker M, Cohen JJ. Effect of substrate-free albumin on perfused rat kidney function. Renal physiology. 1988;10(6):352–60. Epub 1988/01/01. . 3231893 10.1159/000173144

[46] Wegman DH, Apelqvist J, Bottai M, Ekstrom U, Garcia-Trabanino R, Glaser J, et al Intervention to diminish dehydration and kidney damage among sugarcane workers. Scandinavian journal of work, environment & health. 2017 Epub 2017/07/12. doi: 10.5271/sjweh.3659 . 28691728 10.5271/sjweh.3659

[47] Tanriverdi O. A discussion of serum albumin level in advanced-stage hepatocellular carcinoma: a medical oncologist’s perspective. Medical oncology (Northwood, London, England). 2014;31(11):282 Epub 2014/10/16. doi: 10.1007/s12032-014-0282-3 . 25316265 10.1007/s12032-014-0282-3

[48] Mutlu EA, Keshavarzian A, Mutlu GM. Hyperalbuminemia and elevated transaminases associated with high-protein diet. Scandinavian journal of gastroenterology. 2006;41(6):759–60. Epub 2006/05/24. doi: 10.1080/00365520500442625 . 16716979 10.1080/00365520500442625

[49] Kaysen GA, Jones H Jr., Hutchison FN. High protein diets stimulate albumin synthesis at the site of albumin mRNA transcription. Kidney international Supplement. 1989;27:S168–72. Epub 1989/11/01. . 2561516

[50] Lee EH, Kim WJ, Kim JY, Chin JH, Choi DK, Sim JY, et al Effect of Exogenous Albumin on the Incidence of Postoperative Acute Kidney Injury in Patients Undergoing Off-pump Coronary Artery Bypass Surgery with a Preoperative Albumin Level of Less Than 4.0 g/dl. Anesthesiology. 2016;124(5):1001–11. Epub 2016/02/19. doi: 10.1097/ALN.0000000000001051 . 26891150 10.1097/ALN.0000000000001051

[51] Delaney AP, Dan A, McCaffrey J, Finfer S. The role of albumin as a resuscitation fluid for patients with sepsis: a systematic review and meta-analysis. Critical care medicine. 2011;39(2):386–91. Epub 2011/01/21. doi: 10.1097/CCM.0b013e3181ffe217 . 21248514 10.1097/CCM.0b013e3181ffe217

[52] Wiedermann CJ, Dunzendorfer S, Gaioni LU, Zaraca F, Joannidis M. Hyperoncotic colloids and acute kidney injury: a meta-analysis of randomized trials. Critical care (London, England). 2010;14(5):R191 Epub 2010/10/30. doi: 10.1186/cc9308 . 21029460 10.1186/cc9308 PMC3219298

