# Supplementary material for: U-shape association of serum albumin level and acute kidney injury risk in hospitalized patients
Source: PLoS One. 2018 Jun 21;13(6):e0199153. doi: 10.1371/journal.pone.0199153 (PMC6013099; doi:10.1371/journal.pone.0199153)
Supplement: S1 Table — (DOCX) [file pone.0199153.s001.docx]

**S1 Table:** ICD-9 for end-stage renal disease

| ICD-9 | Condition |
| --- | --- |
| 585.5 | Chronic Kidney Disease, stage V |
| 585.6 | End Stage Renal Disease |
| 996.73 | Other complications due to renal dialysis device implant and graft |
| 996.68 | Infection and inflammatory reaction due to peritoneal dialysis catheter |
| 996.56 | Mechanical complication due to peritoneal dialysis catheter |
| 792.5 | Cloudy (hemodialysis or peritoneal dialysis) effluent |
| 458.21 | Hypotension of dialysis |
