# Supplementary material for: U-shape association of serum albumin level and acute kidney injury risk in hospitalized patients
Source: PLoS One. 2018 Jun 21;13(6):e0199153. doi: 10.1371/journal.pone.0199153 (PMC6013099; doi:10.1371/journal.pone.0199153)
Supplement: S2 Table — (DOCX) [file pone.0199153.s002.docx]

**S2 Table:** ICD-9 for principal diagnosis

| Principal diagnosis group | ICD – 9 code |
| --- | --- |
| Cardiovascular disease | 390-459 |
| Hematology/oncology | 280-289, 140-239 |
| Infectious disease | 001-139 |
| Endocrine and metabolic disease | 240-279 |
| Respiratory disease | 460-519 |
| Gastrointestinal disease | 520-579 |
| Injury and poisoning | 800-999 |
| Other | 290-319,320-359,360-389,580-629,630-679,680-709,710-739,740-759,760-779,780-799 |
