# Supplementary figures and images for: U-shape association of serum albumin level and acute kidney injury risk in hospitalized patients
Source: PLoS One. 2018 Jun 21;13(6):e0199153. doi: 10.1371/journal.pone.0199153 (PMC6013099; doi:10.1371/journal.pone.0199153)

**S1 Figure**: Study flow


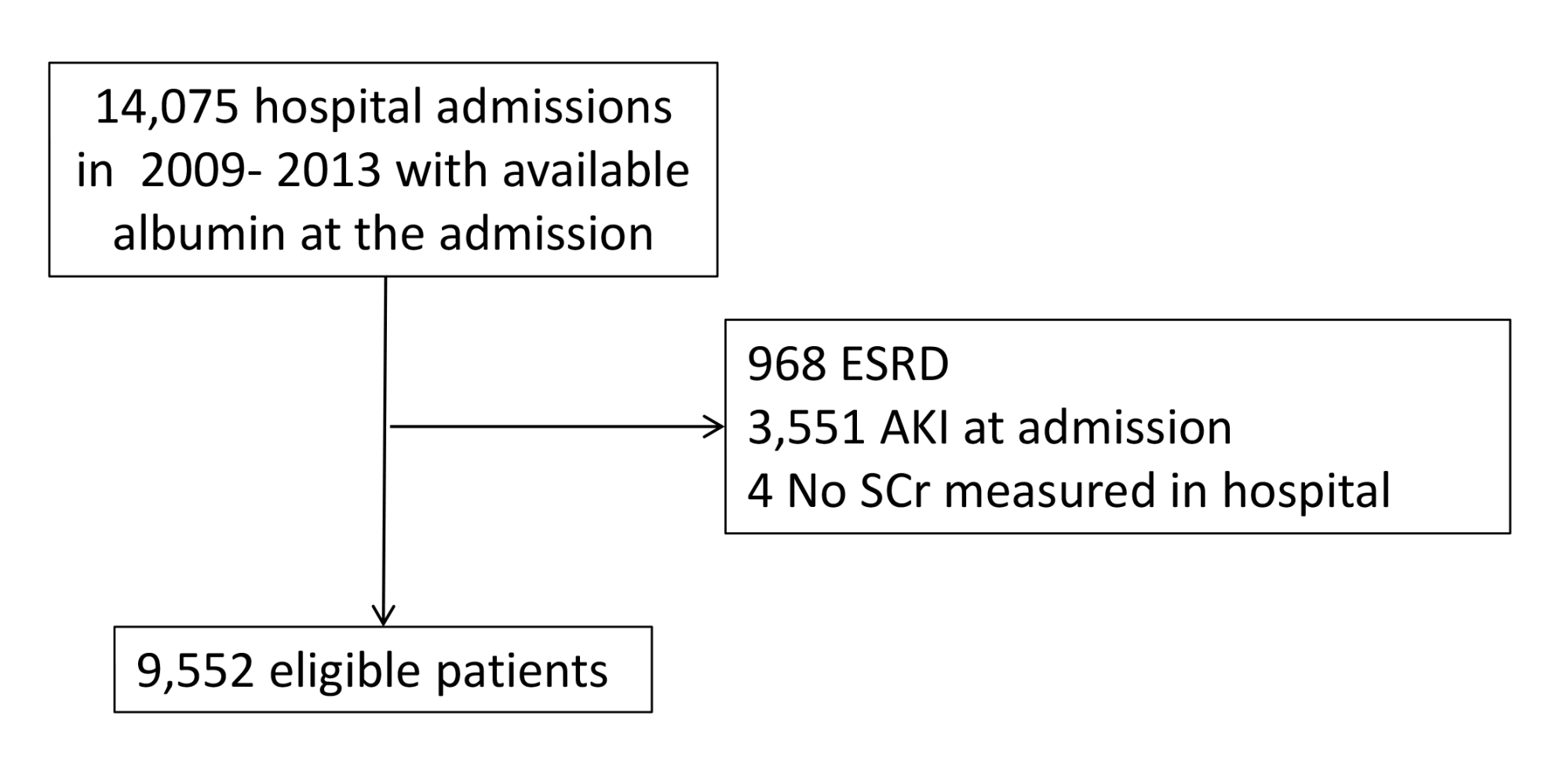

Supplement: S1 Fig — (DOCX) [file pone.0199153.s003.docx]
